# Supplementary material for: scTopoGAN: unsupervised manifold alignment of single-cell data
Source: Bioinform Adv. 2023 Nov 24;3(1):vbad171. doi: 10.1093/bioadv/vbad171 (PMC10701792; doi:10.1093/bioadv/vbad171)
Supplement: vbad171_Supplementary_Data [file vbad171_supplementary_data.pdf]

Supplementary data

scTopoGAN: unsupervised manifold alignment of single-cell data

Akash Singh[1], Kirti Biharie[1,2,3], Marcel J.T. Reinders[1,2,3], Ahmed Mahfouz[1,2,3] and Tamim Abdelaal[1,2,4*]

[1]Delft Bioinformatics Lab, Delft University of Technology, 2628 XE Delft, The Netherlands

[2]Leiden Computational Biology Center, Leiden University Medical Center, 2333ZC Leiden, The Netherlands

[3]Department of Human Genetics, Leiden University Medical Center, 2333ZC Leiden, The Netherlands

[4]LKEB, Department of Radiology, Leiden University Medical Center, 2333ZC Leiden, The Netherlands

*To whom correspondence should be addressed.

Contact: t.r.m.abdelaal@lumc.nl

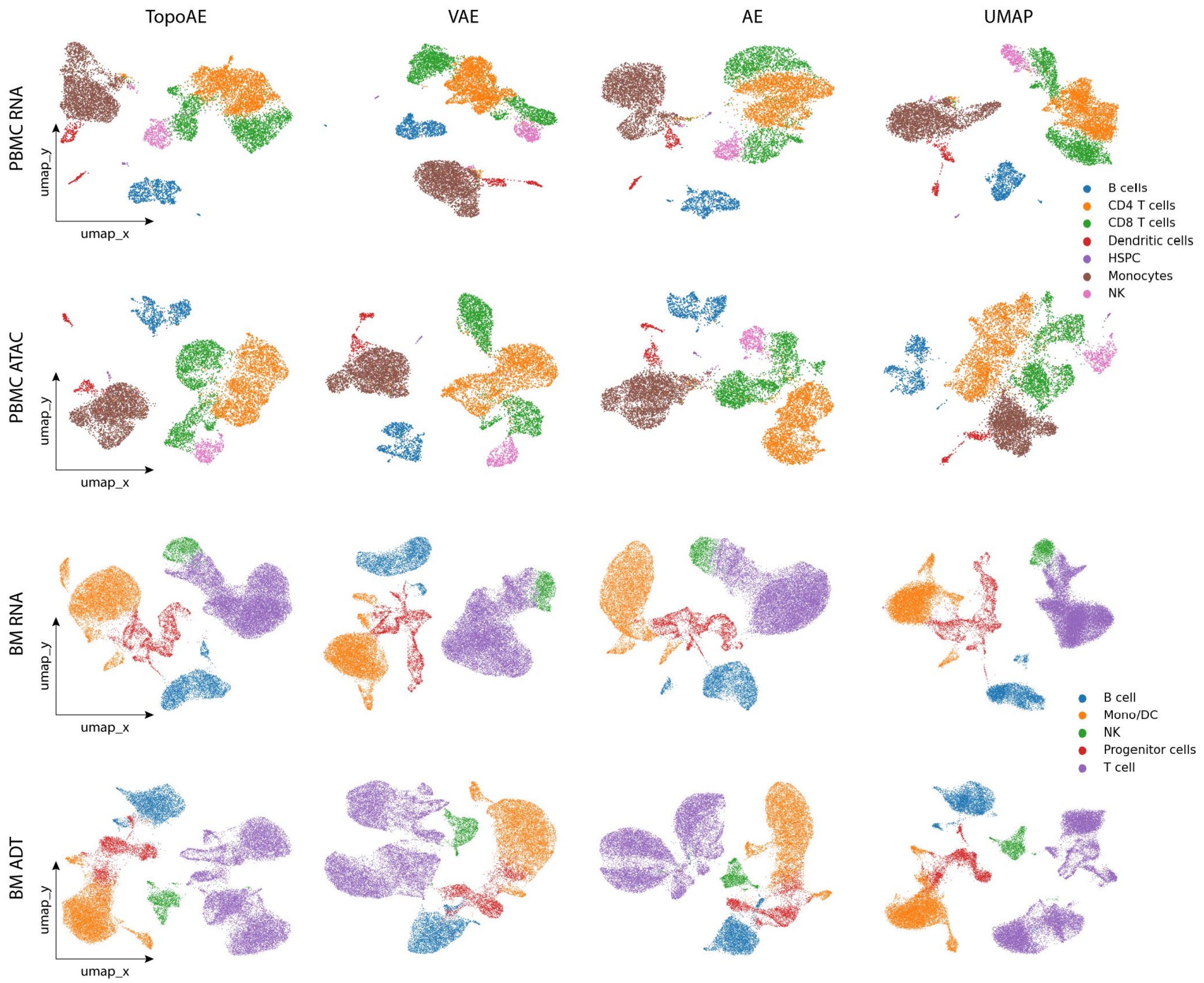

Supplementary Fig. S1 Qualitative comparison of the manifold projection. Plots showing two-dimensional UMAP embeddings of the 8-dimensional manifolds obtained using topoAE, VAE and AE, together with the 2-dimensional embedding obtained using UMAP, for the RNA and ATAC modalities of the Full PBMC dataset (top two rows) and the RNA and ADT modalities of the BM dataset (bottom two rows). All plots are colored according to the cell classes.

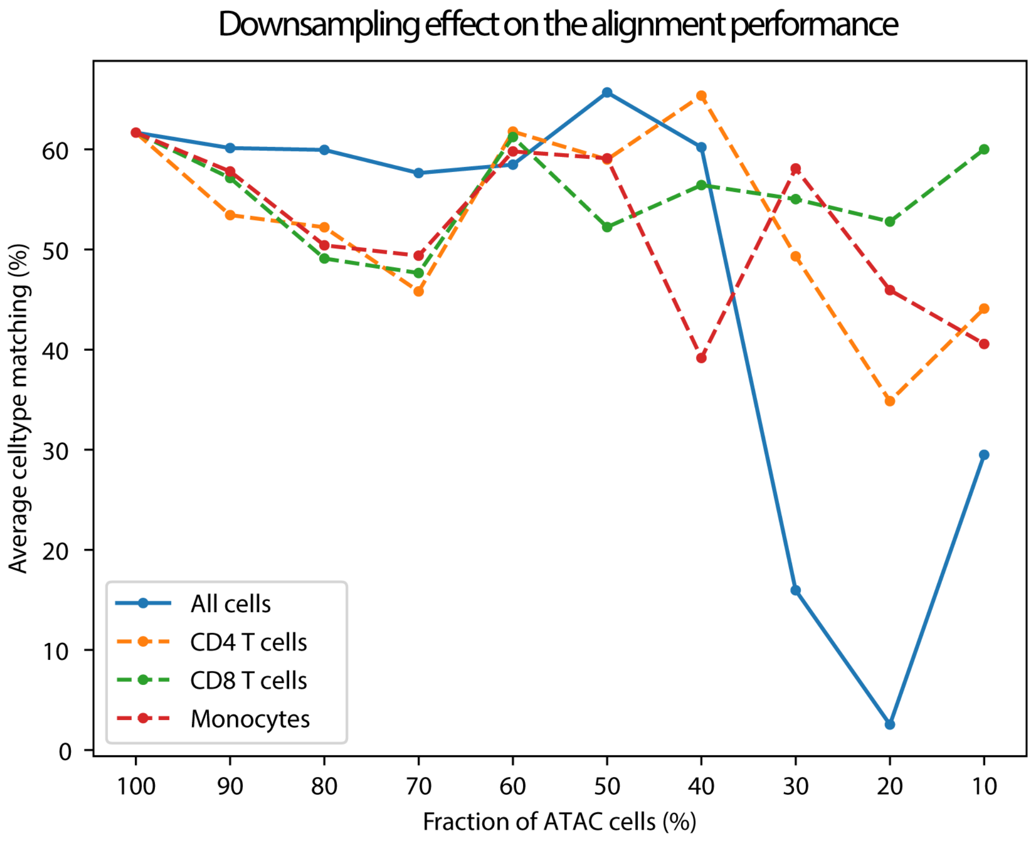

Supplementary Fig. S2 Line plots showing the effect of downsampling the ATAC cells from the Full PBMC data on the alignment performance measured using the average celltype matching score.

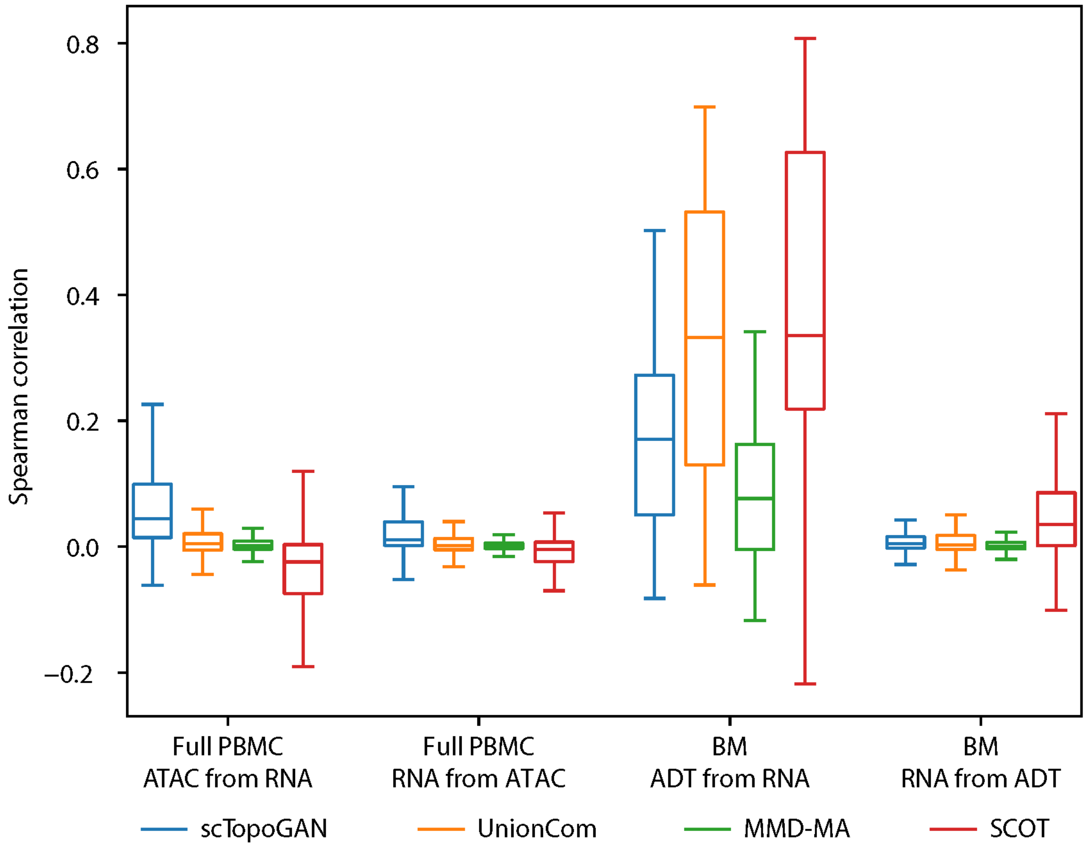

Supplementary Fig. S3 Boxplot showing the Spearman correlation for the cross-modality prediction performed using the aligned Full PBMC and BM datasets by each method. Based on the median Spearman correlation, scTopoGAN and SCOT are the top performing methods for the Full PBMC and the BM datasets, respectively.

Supplementary Table S1 Computational memory and time requirements for each method

Dataset	Method	Average memory usage (GB)	Average run time (minutes)
Full PBMC	scTopoGAN	**2.5**	114
	UnionCom	7.4	90
	MMD-MA	3.0	126
	SCOT	17.9	**20**
Partial PBMC	scTopoGAN	**2.5**	84
	UnionCom	4.6	36
	MMD-MA	2.6	60
	SCOT	8.3	**7**
BM	scTopoGAN	**2.5**	144
	UnionCom	6.4	108
	MMD-MA	3.0	120
	SCOT	16.6	**30**
